# Half logistic-truncated exponential distribution: Characteristics and applications

**DOI:** 10.1371/journal.pone.0285992

**Published:** 2023-11-14

**Authors:** Ahtasham Gul, Amjad Javaid Sandhu, Muhammad Farooq, Muhammad Adil, Yasir Hassan, Faridoon Khan

**Affiliations:** 1 Pakistan Bureau of Statistics, Islamabad, Pakistan; 2 Department of Statistics, COMSATS University Islamabad, Lahore Campus, Islamabad, Pakistan; 3 Lahore Business School, The University of Lahore, Lahore, Pakistan; University of Porto Faculty of Engineering: Universidade do Porto Faculdade de Engenharia, PORTUGAL

## Abstract

Gul and Mohsin 2021 developed a new modified form of renowned “Half logistic” distribution introduced by Balakrishnan (1991) and named it half logistic-truncated exponential distribution (HL-TEXPD). Some mathematical characteristics are studied, including hazard function, P^*th*^ percentile, moment generating function and Shannon entropy. Simulation study is performed to examine the behaviour of parameter estimates. The proposed model is fitted on three real data sets to check its efficacy. Additionally, TTT (total time on test) plot is drawn to study the failure rate of the three data sets. The results verdict that HL-TEXPD can be efficiently utilized in the field of engineering and medical sciences based on the data sets under study contrary to the classical and baseline models.

## 1 Introduction

Half logistic distribution (HLD) is one of well-known lifetime models originated by [1] has been used in reliability analysis by many researchers. Half logistic distribution (HLD) is the absolute value of logistic distribution. The simplicity of HLD attracted many researchers to study its various characteristics.

Since the emergence of half logistic distribution, several generalizations have been introduced. [2] developed Power half logistic distribution using the power transformation algorithm for modeling the three data sets of engineering sciences, i.e. **i**. number of revolutions (in millions) of 23 ball bearings before their failure; **ii**. strengths of glass fibers and **iii**. camber of 497 lead wires in the manufacturing of miniature radio tubes. [3] developed Extended half logistic distribution to study the estimated lifetime of an electronic device by using several estimation algorithms. [3] showed that maximum likelihood method is best technique for parameter estimation when sample size is small contrary to weighted least square method which is suitable for large sample size. [4] suggested a new generator to develop the Exponentiated half-logistic family of distributions having additional two shape parameters. The worth of Exponentiated half-logistic model is portrayed by fitting it on two real data sets, i.e. first data is consisting of the survival times of guinea pigs (a pocket pet) and in second data set Exponentiated half-logistic model is fitted to measure the quantity of Carbon Monoxide(CO) in several brands of cigarettes. [4] also introduced the bivariate version of Exponentiated half-logistic family. [5] introduced McDonald half-logistic distribution, that is a generalization of half-logistic distribution. [6] generated type-I half logistic Burr-X density function for modeling the two different engineering data i.e. to model the strengths of 63 glass fibers obtained from employees working at National Physical Laboratory situated in UK and life of fatigue fracture of Kevlar 373/epoxy. [7] derived recursive relations of moments for half-logistic distribution and computed first moment, variances and covariances of order statistics by using these relations. [8] computed the best linear unbiased estimates (BLUE) of the location and scale parameters for half-logistic distribution. [9] used the half-logistic model to obtain maximum likelihood and average maximum likelihood estimates for the scale parameter *σ* under progressive Type-II censored samples, and compared mean squared error (MSE) of the suggested estimators. [10] applied generalized half-logistic distribution to derive the entropy for Type-II censored samples. [11] studied the characterization properties of half-logistic model.

Exponential distribution is widely used in multidimensional areas, for instance, modeling the lifetimes of manufactured items ([12, 13]) or remission times in chronic diseases([14]). Exponential distribution attracts the researchers due to its worthy property called “memory less property”, i.e.

P{Y≤(y+z|Y>z)}=P(Y≤y),

Analogous to half-logistic distribution, several generalized families of exponential distribution have so far been developed by the researchers. Some of these well known generalizations are: Beta exponential distribution by [15], Exponentiated exponential family by [16] and Generalized exponential distribution by [17]. [18] introduced Erlang-Truncated exponential (ETE) and Binomial-exponential distribution by using the mixture of distributions technique. [19] derived the recurrence relations for moments of generalized order statistics using Erlang-Truncated Exponential model. [20] introduced an extension of Erlang-Truncated exponential (ETE) called it Extended Erlang-Truncated exponential distribution (EE-TED) and fitted it on uncensored rain data. The author revealed that EE-TED better fit the rainfall data than Erlang-Truncated Exponential (ETE) distribution and other three competing models.

The application of exponential distribution is very prominent in censored data. [21] derived the recursive relations of progressive type II right censored order statistics for exponential and Truncated exponential distributions. [22] derived Single and product moments of order statistics and computed the three estimators i.e maximum likelihood (MLE), best linear unbiased (BLUE) and uniformly minimum variance unbiased (UMVUE) of location and scale parameters for exponential, truncated exponential and two-parameter exponential distributions.

Statistical modeling plays a vital role in every field of life, specifically in probability theory, which is often used to figure out the variation and to make inferences based on observed data. In many scenarios, the parent distribution neither properly describes nor fit the data in better form. Thus this situation gives the space and motivation to researchers for the development of new statistical models. As a result, in recent decades, numerous new generalized distributions have been established and getting more importance. The main purpose behind developing these distributions is that they have more parameters. According to [23], the fitting of four parameter distributions is sufficient for most practical phenomenon.

The literature shows that the truncated distributions so far are developed by using the subjective approach, i.e. the researchers selected threshold point on their own choice and no methodology or mathematical algorithm has not been developed for generating family of truncated distributions alike beta generated family, Kumaraswamy G family, Exponentiated family or T-X family of distributions, etc.

Similarly, in real life situation when we face complex scenario, simple probability distributions remain unable to model them, instead a nested model over restricted domain is more appropriate. The outcome which depends on other restricted phenomenon rather than simple input variable, the truncated distribution undeniably can assist the researcher to make more precise prediction. In contrast to existing truncated distributions, our proposed methodology for developing truncated distributions is more flexible. In other words, unlike existing methods, we can use or modify our proposed methodology for specific circumstances. For instance, in USA the minimum legal alcohol drinking age is 21 years and minimum driving license obtaining age is 16 years. If the researcher is interested to investigate all traffic crash fatalities in the United States involving drunk drivers then the threshold will be required to circumvent us from the irrelevant range (0-20) and justly consider those drivers having age 21 years or above, which will obviously improve the predictability.

The core objective of this manuscript is to model finite real data by using truncated finite distribution rather than the models having infinite index. The aforementioned question motivated the authors and in the present paper, we introduced and investigate a novel truncated version of exponential distribution using a transformation of half logistic random variable.

The present manuscript is designed as; HL-TEXPD is introduced and some mathematical characteristics of the proposed distribution are studied in Section 2. In Section 3, HL-TEXPD parameter is estimated by using maximum likelihood estimation technique and the Monte Carlo simulation is performed to study the stability of model parameter. In Section 4, HL-TEXPD is fitted on three data sets. Finally, in Section 5, concluding remarks are recorded.

## 2 Half Logistic-Truncated Exponential Distribution (HL-TEXPD)

[24, 25] suggested a new method for generating a family of truncated distributions called T-*X*_*T*_ family of distributions by using a new function given as:

$$\begin{array}{c} W\left(F\left(x_{T}\right)\right)=-\text{log}\left\{1-F\left(x|x>\tau \right)\right\}. \end{array}$$ (1)

Let X be a non-negative random variable truncated on left having probability density function (pdf) *f*(*x*_*T*_) and distribution function (cdf) *F*(*x*_*T*_) on domain [*τ*, ∞). Also let T be a random variable with pdf r(t) and cdf R(t) on interval [−∞ ≤ *a* ≤ *t* ≤ *b* ≤ ∞).

Then the cdf of T-*X*_*T*_ family of distributions is

$$\begin{array}{c} G\left(x_{T}\right)=\int_{\tau }^{-\text{log}\{1-F(x|x>\tau )\}}r(t)dt, \end{array}$$ (2)

$$\begin{array}{c} G\left(x_{T}\right)=R[-\text{log}\{1-F(x_{T})\}], \end{array}$$ (3)

where R(t) is the cdf of random variable T, while the corresponding pdf of T-*X*_*T*_ family of distributions is

$$\begin{array}{c} g\left(x_{T}\right)=r[-\text{log}\{1-F(x|x>\tau )\;\}]\frac{f(x|x>\tau )\;}{1-F(x|x>\tau )\;},\;x>\tau . \end{array}$$ (4)

$$\begin{array}{c} g\left(x_{T}\right)=r[-\text{log}\{1-F(x_{T})\}]\{\frac{f(x_{T})}{1-F(x_{T})}\}, \end{array}$$ (5)

$$\begin{array}{c} g\left(x_{T}\right)=r[H(x_{T})]h\left[x_{T}\right]. \end{array}$$ (6)

The idea presented in Eq (2) is extended by the method of generating a new family of distributions called T-X family of distributions proposed by [26] which is the extension of Beta Generated distributions originally introduced by [27].

Suppose X be an exponential random variable having density function

$$\begin{array}{c} f\left(x\right)=\theta e^{-\theta x}, \end{array}$$ (7)

with corresponding cdf

$$\begin{array}{c} F\left(x\right)=1-\theta e^{-\theta x}. \end{array}$$ (8)

The T-Truncated Exponential distribution defined by [24] is expressed as:

$$\begin{array}{c} g(x)=\theta r\{\theta (x-a)\}. \end{array}$$ (9)

Suppose T be a half logistic stochastic variable having pdf

$$\begin{array}{c} f\left(t\right)=\frac{2e^{-t}}{{\left(1+e^{-t}\right)}^{2}},\;where\;t>0, \end{array}$$ (10)

and cdf is

$$\begin{array}{c} F\left(t\right)=\frac{1-e^{-t}}{1+e^{-t}}. \end{array}$$ (11)

Thus the resulting Half Logistic-Truncated Exponential distribution (HL-TEXPD) is developed by using (9) and (10) as;

$$\begin{array}{c} g\left(x_{T}\right)=\frac{2\theta e^{-\theta (x-a)}}{{\left\{1+e^{-\theta (x-a)}\right\}}^{2}}\;for\;x>a, \end{array}$$ (12)

cdf of HL-Truncated Exponential Distribution

$$\begin{array}{c} G\left(x_{T}\right)=\frac{1-e^{-\theta (x-a)}}{1+e^{-\theta (x-a)}}. \end{array}$$ (13)

**Fig 1:**
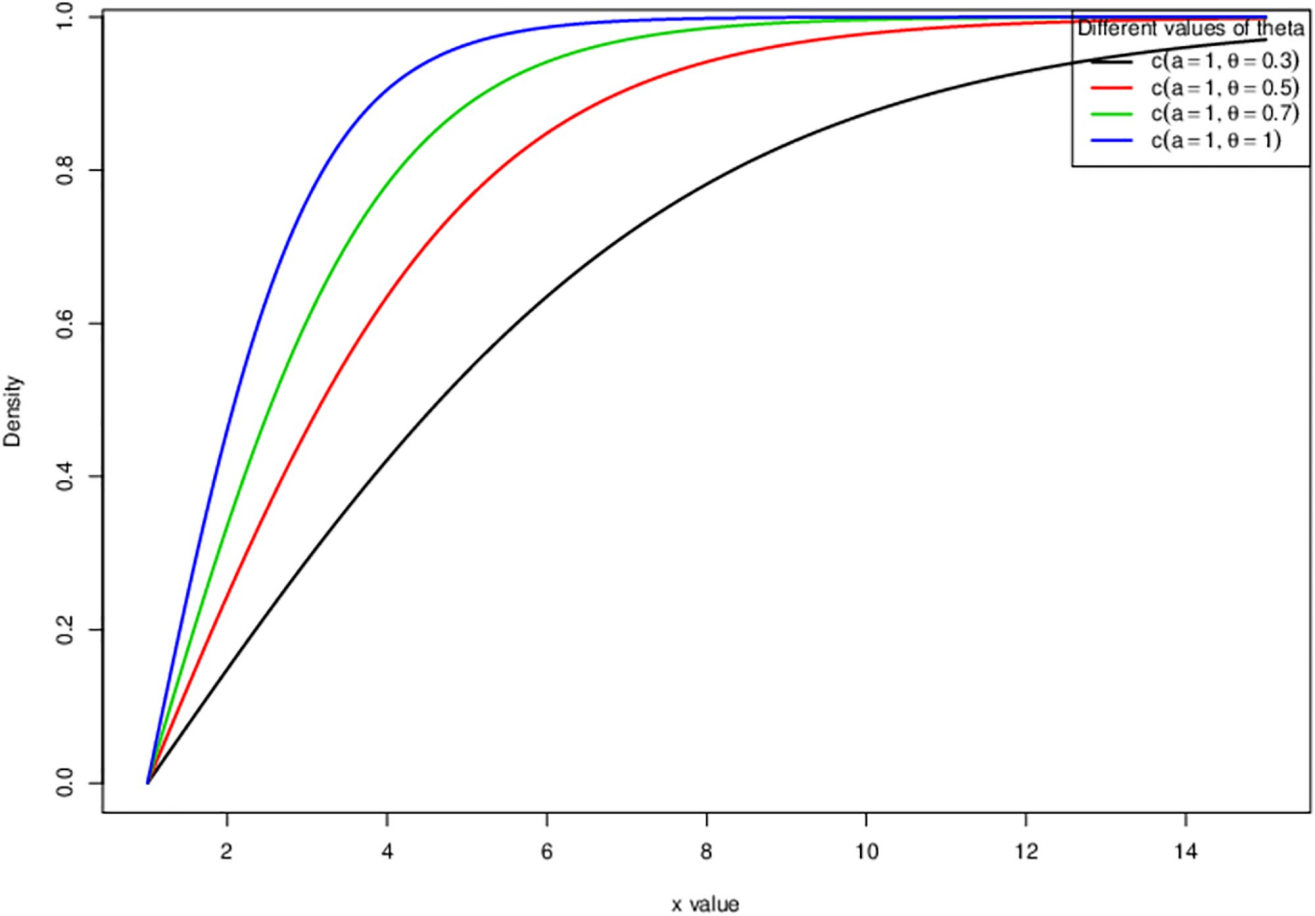
Cumulative distribution function (cdf) of Half logistic-truncated exponential distribution.

**Fig 2:**
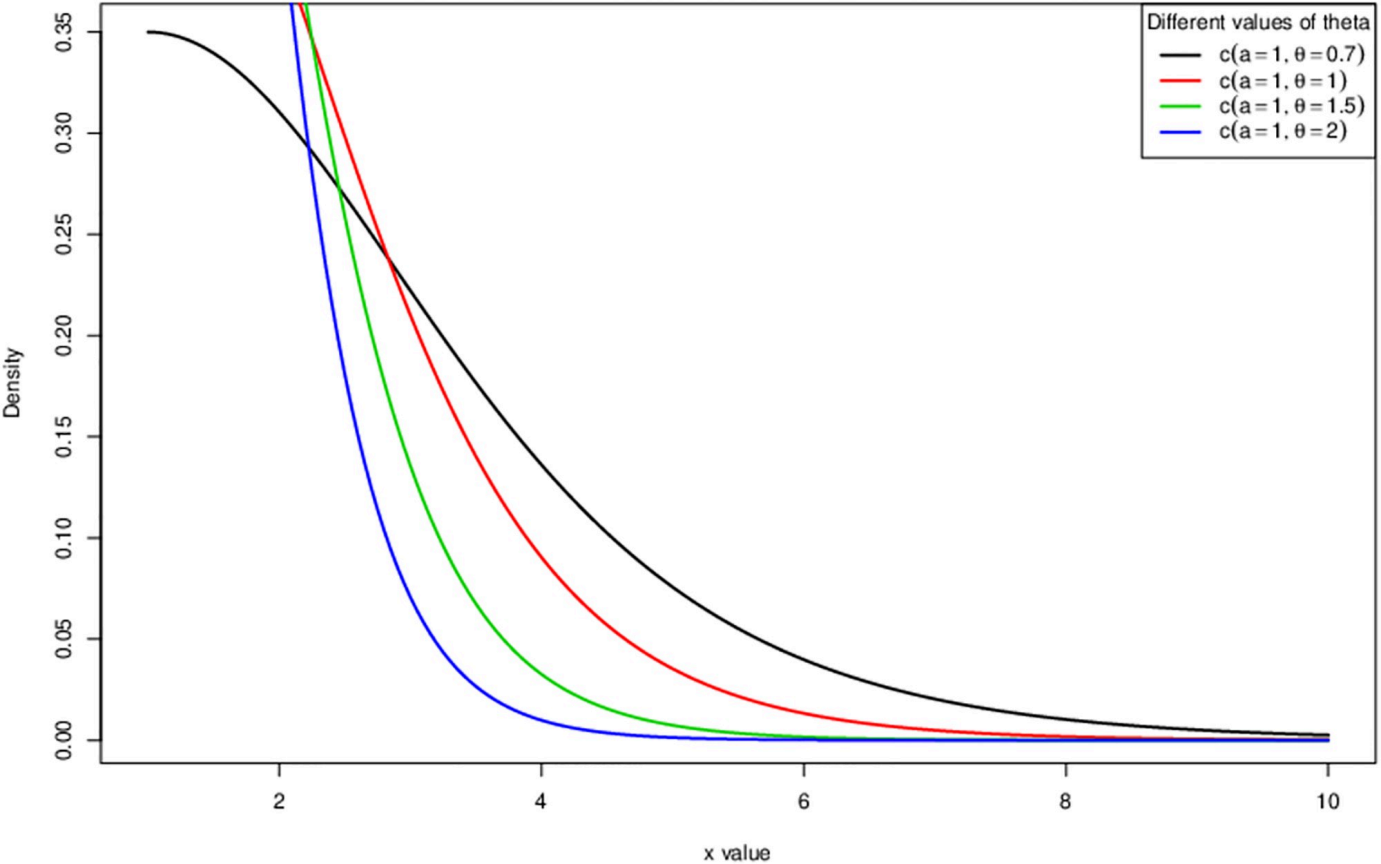
Probability density function (pdf) of Half logistic-truncated exponential distribution.

The Fig 1 is the cumulative density function (cdf) of HL-TEXPD plotted at different values of *θ*. It is observed that the curve holds almost vertical cdf that indicates a high kurtosis distribution with heavy/fatter tail and where the centre of the distribution is pulled up by increasing the value of *θ*. Similarly the above, the above Fig 2 is sketched at different values of *θ* which connotes that by increasing the value of *θ*, the curve becomes leptokurtic i.e. a heavy/fatter tailed resulting in a capturing of extreme values.

### 2.1 Properties of the HL-TEXPD

Some fundamental statistical properties of proposed model are presented in this Section, as follows:

**Lemma 2.0.1**. *Let random variable t is distributed as HL-TEXPD with parameter a and θ, then its hazard function is*

$$h\left(t\right)=\frac{\theta }{1+e^{-\theta (t-a)}}.$$

*Proof*. We define hazard function as

$$\begin{array}{c} h\left(t\right)=\frac{g(t)}{1-G(t)}=\frac{f(t)}{R(t)}=\frac{f(t)}{s(t)}, \end{array}$$ (14)

using (12) and (13), we get

$$\begin{array}{c} h\left(t\right)=\frac{\theta }{1+e^{-\theta (t-a)}} \end{array}$$ (15)

**Fig 3:**
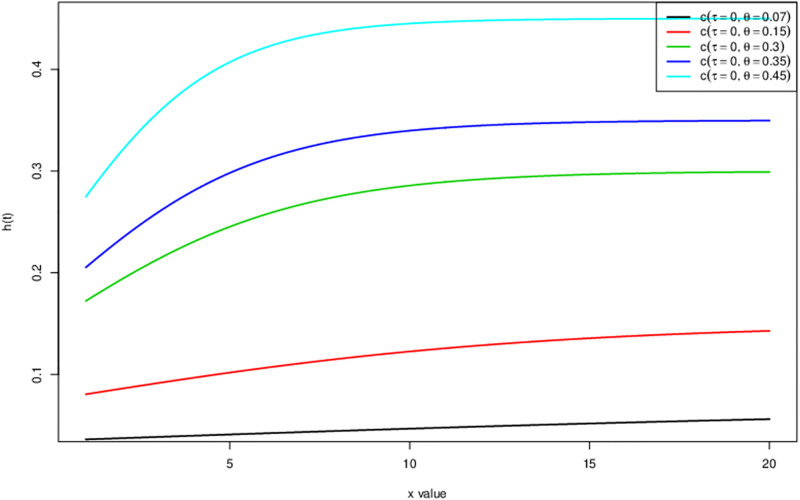
Hazard rate of Half logistic-truncated exponential distribution.

The hazard rate function also known as instantaneous failure rate or force of mortality is the probability of the event occurring during any given time point. The Fig 3 is the hazard rate h(t) of HL-TEXPD sketched at different values of *θ*. We can conjecture from Fig 3 that hazard rate is monotonically increasing at different values of *theta*.

**Lemma 2.0.2**. *Let X_T_ be a random variable following HL-TEXPD, then its P^th^ percentile is*

$$x_{T}=a-\text{log}(\frac{1-p}{1+p}{)}^{1/\theta }.$$

*Proof*. The P^*th*^ percentile can be used to compute median and fractile. It can also be used to generate random numbers.

$$G(x_{T})=P.$$

$$\frac{1-e^{-\theta (x_{T}-a)}}{1+e^{-\theta (x_{T}-a)}}=P,$$

$$\begin{array}{c} x_{T}=a-\text{log}(\frac{1-p}{1+p}{)}^{1/\theta }. \end{array}$$ (16)

**Theorem 2.1**. *If X_T_ follows the HL-TEXPD, then the Shannon’s entropy is*

$$\eta_{x_{T}}=1.6989-\text{log}\left(\theta \right).$$

*Proof*. Shannon’s Entropy is a measure of uncertainty (or variability) associated with random variables.

$$\eta_{x_{T}}=-E[\text{log}\{g\left(x_{T}\right)\}\;]$$

$$H=-\int_{a}^{\infty }\{\text{log}\;g(x_{T})\}g\left(x_{T}\right)dx_{T}\;\;\;,$$

$$\begin{array}{c} \eta_{x_{T}}=-E[\text{log}\{\frac{2\theta e^{-\theta (x_{T}-a)}}{{\left\{1+e^{-\theta (x_{T}-a)}\right\}}^{2}}\}\;], \end{array}$$ (17)

$$\eta_{x_{T}}=-E\left\{\text{log}\left(2\right)\right\}-E\left\{\text{log}\left(\theta \right)\right\}+\theta E\left(x_{T}-a\right)+2E\left\{\text{log}\left(1+e^{-\theta (x_{T}-a)}\right)\right\},$$

$$\begin{array}{c} \eta_{x_{T}}=-\text{log}\left(2\right)-\text{log}\left(\theta \right)+\theta E\left(x_{T}-a\right)+\;\;2E\left\{\text{log}\left(1+e^{-\theta (x_{T}-a)}\right)\right\}, \end{array}$$ (18)

$$I_{1}=E\left(x_{T}-a\right),$$

$$I_{1}=\int_{a}^{\infty }x_{T}\;\;g\left(x_{T}\right)dx_{T}-a\int_{a}^{\infty }g\left(x_{T}\right)dx_{T},$$

$$\begin{array}{c} I_{1}=\frac{\text{log}(4)}{\theta }, \end{array}$$ (19)

Now

$$I_{2}=E\left\{\text{log}\left(1+e^{-\theta (x_{T}-a)}\right)\right\},$$

$$I_{2}=\int_{a}^{\infty }\text{log}\{1+e^{-\theta (x_{T}-a)}\}\frac{2\theta e^{-\theta (x_{T}-a)}}{{\left\{1+e^{-\theta (x_{T}-a)}\right\}}^{2}}dx_{T},$$

let

$$1+e^{-\theta (x_{T}-a)}=u,then$$

$$x=a-\frac{\text{log}(u-1)}{\theta }\;and\;dx=-\frac{1}{\theta (u-1)}du,$$

$$I_{2}=\int_{1}^{2}\frac{\text{log}\;u}{u^{2}}du.$$

$$\begin{array}{c} I_{2}=1-\text{log}\left(2\right), \end{array}$$ (20)

using (19) and (20) in (18), we get

$$\eta_{x_{T}}=-\text{log}\left(2\right)-\text{log}\left(\theta \right)+\theta \frac{\text{log}(4)}{\theta }+2\{1-\text{log}(2)\},$$

$$\begin{array}{c} \eta_{x_{T}}=1.6989-\text{log}\left(\theta \right). \end{array}$$ (21)

**Theorem 2.2**. *The P^th^ raw moment of HL-TEXPD is given by*

$$\begin{array}{c} E\left(x_{T}^{p}\right)=2\int_{0}^{\infty }\frac{{\left(a+\frac{u}{\theta }\right)}^{p}\;e^{-u}}{{\left\{1+e^{-u}\right\}}^{2}}du. \end{array}$$ (22)

*Proof*. By definition

$$E\left(x_{T}^{p}\right)=2\theta \int_{a}^{\infty }\frac{x^{p}\;e^{-\theta (x-a)}}{{\left\{1+e^{-\theta (x-a)}\right\}}^{2}}dx,$$

$$x=a+\frac{u}{\theta },$$

$$\begin{array}{c} E\left(x_{T}^{p}\right)=2\int_{0}^{\infty }\frac{{\left(a+\frac{u}{\theta }\right)}^{p}\;e^{-u}}{{\left\{1+e^{-u}\right\}}^{2}}du. \end{array}$$ (23)

The expression in (23) is used to compute the non-central moments for HL-TEXPD.

**Corollary 2.2.1**. *If X_T_ follows HL-TEXPD, then the First 4 raw moments can be obtained using* (23)

*Proof*. For convince, we used the mathematical package [28] ver.9.0.1.0 and putting P = 1, 2, 3, 4 in (23), we get

$$E\left(x_{T}^{1}\right)=a+\frac{\text{log}(4)}{\theta },$$

$$\begin{array}{c} E\left(x_{T}^{1}\right)=a+\frac{1.38629}{\theta }, \end{array}$$ (24)

$$\begin{array}{c} E\left(x_{T}^{2}\right)=\frac{\pi^{2}+3a\theta \left\{a\theta +\text{log}\left(16\right)\right\}}{3\theta^{2}}, \end{array}$$ (25)

$$\begin{array}{c} E\left(x_{T}^{3}\right)=\frac{a\theta [\pi^{2}+a\theta \left\{a\theta +\text{log}\left(64\right)\right\}+9zeta\left(3\right)]}{\theta^{3}}, \end{array}$$ (26)

$$\begin{array}{c} E\left(x_{T}^{4}\right)=\frac{7\pi^{4}}{15\theta^{4}}+\frac{a^{2}\theta \left[2\pi^{2}+a\theta \left\{a\theta +\text{log}\left(256\right)\right\}+36zeta\left(3\right)\right]}{\theta^{3}}, \end{array}$$ (27)

**Corollary 2.2.2**. *If X_T_ be a HL-TEXPD stochastic variable, then variance, skewness and kurtosis can be computed using* (24) *to* (27)

*Proof*. Since

$$\sigma_{x}^{2}=E{\left\{x-E(x)\right\}}^{2}$$

$$var\left(x_{T}\right)=\frac{\pi^{2}-3\;\text{log}\;[4{]}^{2}}{3\theta^{2}},$$

$$\begin{array}{c} var\left(x_{T}\right)=\frac{1.368}{\theta^{2}} \end{array}$$ (28)

$$u_{3}=m_{3}-3m_{1}m_{2}+2m_{1}^{3}$$

$$\begin{array}{c} u_{3}=\frac{-\pi^{2}\;\text{log}\;[4]+2\;\text{log}\;[4{]}^{3}+a\theta (6\;\text{log}\;[4{]}^{2}-\;\text{log}\;[16]\;\text{log}\;[64])+9Zeta[3]}{\theta^{3}}, \end{array}$$ (29)

$$\begin{array}{c} u_{3}=\frac{2.464}{\theta^{3}}. \end{array}$$ (30)

$$u_{4}=m_{4}-4m_{1}m_{3}+6m_{1}^{2}m_{2}-3m_{1}^{4},$$

$$u_{4}=[\begin{array}{c} a^{4}+\frac{7\pi^{4}}{15\theta^{4}}+\frac{2a^{2}\pi^{2}}{\theta^{2}}-3(a+\frac{\text{log}[4]}{\theta }{)}^{4}+6(a+\frac{\text{log}[4]}{\theta })(a^{2}+\frac{\pi^{2}}{3\theta^{2}}+\frac{a\;\text{log}\;[16]}{\theta })\\ +\frac{a^{3}\;\text{log}\;[256]}{\theta }+\frac{36aZeta[3]}{\theta^{3}}-4(a+\frac{\text{log}[4]}{\theta })(a^{3}+\frac{a\pi^{2}}{\theta^{2}}+\frac{a^{2}\;\text{log}\;[64]}{\theta }+\frac{9Zeta[3]}{\theta^{3}}) \end{array}],$$

$$\begin{array}{c} u_{4}=\frac{\left[\begin{array}{c} -25.613+(27.364-86.69a)\theta +(42.800-77.393a)a\theta^{2}\\ +(24.953-33.27a)a^{2}\theta^{3}+(6-6a)a^{3}\theta^{4} \end{array}\right]}{\theta^{4}}. \end{array}$$ (31)

$$Skewness=\frac{\mu_{3}}{{\left(\mu_{2}\right)}^{3/2}},$$

$$\begin{array}{c} Skewness=1.54\sqrt{\theta }. \end{array}$$ (32)

$$Kurtosis=\frac{\mu_{4}}{{\left(\mu_{2}\right)}^{2}}$$

$$Kurtosis=\frac{\left[\begin{array}{c} 7\pi^{4}-3{\left(a+\frac{\text{log}(4)}{\theta }\right)}^{4}+\frac{2{\left(a+\frac{\text{log}(4)}{\theta }\right)}^{2}\left[\pi^{2}+3a\theta \left\{a\theta +\text{log}\left(16\right)\right\}\right]}{\theta^{2}}\\ -\frac{4(a+\frac{\text{log}(4)}{\theta })a\theta \left[\pi^{2}+a\theta \left\{a\theta +\text{log}\left(64\right)\right\}+9zeta\left(3\right)\right]}{\theta^{3}}+\frac{a^{2}\theta \left[2\pi^{2}+a\theta \left\{a\theta +\text{log}\left(256\right)\right\}+36zeta\left(3\right)\right]}{\theta^{3}} \end{array}\right]}{{\left\{\frac{\pi^{2}+3a\theta \left\{a\theta +\text{log}\left(16\right)\right\}}{3\theta^{2}}-{\left(a+\frac{\text{log}(4)}{\theta }\right)}^{2}\right\}}^{2}}.$$

## 3 Parameter estimation of HL-TEXPD distribution

Here, the parameter estimates of HL-TEXPD are computed by using Maximum Likelihood Estimation (MLE) technique. The log-likelihood function is defined as:

$$\text{log}\;L\left(a,\Theta ;x_{T}\right)=\text{log}\prod_{i=1}^{n}g\left(x_{T}\right)$$

$$\text{log}\;L\left(a,\theta ;x_{T}\right)=n\;\text{log}\;\;(2)+n\;\text{log}(\theta )-\theta \sum_{i=1}^{n}\left(x_{i}-a\right)\;\;-2\sum_{i=1}^{n}\;\text{log}\{1+e^{-\theta (x_{i}-a)}\},$$

$$\begin{array}{c} \hat{a}=min\left[x_{i}\right]. \end{array}$$ (33)

$$\begin{array}{c} \frac{\text{log}\;L(a,\theta ;x_{T})}{\partial \theta }=\frac{n}{\theta }-\;\sum_{i=1}^{n}\left(x_{i}-a\right)\;+2\;\sum_{i=1}^{n}\frac{\left(x_{i}-a\right)e^{-\theta (x_{i}-a)}}{1+e^{-\theta (x_{i}-a)}}. \end{array}$$ (34)

### 3.1 Simulation study

Here, the Monte Carlo simulation is performed to study the stability of model parameters. The simulation is run 1000 times for four different combinations of the parameter to draw the random samples of size n each from the HL-TEXPD (*θ*). The model parameter is estimated by using ML estimation method.

Table 1 presents maximum likelihood estimates (MLE), average ML estimates of the parameter with standard errors (SEs), biases, mean square errors (MSEs), mean relative errors (MRE) and corresponding 99% coverage probability for approximate confidence intervals for samples of sizes 20, 50, 100 and 200. A fixed seed is used to generate such random numbers, implying that all results of these studies can always be exactly replicated. The Monte Carlo simulation is performed in the following two steps:

1. generate one thousand samples of size n = 20, 50, 100 and 200 each using (21).
2. compute the average MLEs, the average ML estimates, SEs, MSEs, MREs and CIs for each sample, i.e. ($\hat{\theta }$).
  The bias and the MSE are computed by using
  $$\text{bias}=\frac{1}{1000}\sum_{i=1}^{1000}({\hat{\alpha }}_{i}-\alpha ),$$
  $$\text{MSE}=\frac{1}{1000}\sum_{i=1}^{1000}(\hat{\sigma_{\alpha_{}}^{2}}+\{bias({\hat{\alpha }}_{i}){\}}^{2}),$$
  and
  $$\text{MRE}=\frac{1}{1000}\sum_{i=1}^{1000}(\theta -\hat{\theta }/\theta ),$$
  respectively.
3. Similarly, the two sided asymptotic (1-*ε*)% CI for the parameter *α* is computed by using
  $$\hat{\alpha }\;\pm Z_{\varepsilon /2}\sqrt{\frac{{\hat{\sigma }}_{\alpha }^{2}}{1000}},\;$$
  where *Z*_*ε*_ represents (1-*ε*)% percentile of the standardized normal distribution.
4. The simulated coverage probabilities for two-sided approximate at 99% confidence intervals Pr($\theta \in \hat{I}$) (The parameter of interest *θ* is estimated by $\hat{\theta }$ and the confidence interval $\hat{I}$) based on the normal-approximate distribution are computed.

**Table 1:** MLE, average estimated values, corresponding SEs (given in parentheses) Bias, MSE, MRE and 99% coverage probabilities of model parameters for HL-TEXPD.

Actual Values	n	MLE	Average Estimate (S.E)	Bias	MSE	MRE	99% coverage probability
*θ* = **0.5**	**20**	-37.42679	0.58177 (0.00025)	0.08177	0.00669	0.16353	35%
	**50**	-97.82035	0.55785 (0.00013)	0.01786	0.00435	0.10185	52%
	**100**	-192.12907	0.53604 (0.00011)	0.01008	0.00178	0.06610	75%
	**200**	-415.46122	0.50456 (0.00001)	0.00456	0.00101	0.04988	90%
*θ* = **0.3**	**20**	-56.61272	0.22155 (0.078453)	-0.078453	0.69930	0.18037	24%
	**50**	-124.0992	0.3114 (0.00004)	0.0113	0.0017	0.1057	79%
	**100**	-249.7075	0.30523 (0.00002)	0.00523	0.00071	0.06894	93%
	**200**	-500.90730	0.30253 (0.00001)	0.00253	0.00033	0.04794	99%
*θ* = **0.05**	**20**	-84.61939	0.05480 (0.00001)	0.00480	0.00014	0.17783	37%
	**50**	-213.7457	0.05745 (0.00001)	0.00174	0.00004	0.09905	68%
	**100**	-429.028	0.05079 (0.00001)	0.00079	0.00005	0.06768	76%
	**200**	-859.524	0.05038 (0.00001)	0.00036	0.00001	0.04779	95%

Table (1) shows that biases and MSEs fluctuate with respect to n. It is observed from Table 1 that AEs of HL-TEXPD parameter approaches the true values of the parameter as n increases. The biases and MSEs for parameter approaches to zero as sample size increases. Moreover, the simulation results connote that 99% coverage probability for approximate confidence intervals of true parameter based on MLEs give satisfactory results. These findings endorse the asymptotic theory (large sample) of the normal distribution showing that the errors of these estimates, as expected, decrease when n increases.

## 4 Real life application

We demonstrate the performance of the HL-TEXPD by fitting on three data sets relating to engineering and actuarial sciences. The unknown parameters of HL-TEXPD are calculated using ML estimation, Akaike Information Criterion (AIC), and Bayesian Information Criterion (BIC). The smallest values of these measures indicate that the model better fits the data. The AIC and BIC for a model are defined as:

AIC = 2k-2log*(likelihood)*,

where

k is total number of parameters and *(likelihood)* is actually L(Θ; *x*_*T*_).

and

BIC = klog*(n)*-2log*(likelihood)*,

where

k is the number of parameters in the statistical model and n denotes the sample size.

### 4.1 Application 1: Mechanical engineering

To emphasize the eminence of HL-TEXPD, we fitted on data consisting the life of fatigue fracture of Kevlar 373/epoxy at fixed pressure until all had failed. [29] has already fitted the model on same data set. The data set are: 0.0251, 0.0891, 0.0886, 0.3113, 0.8425, 0.2501, 0.4763, 0.5650, 0.5671, 0.3451, 0.8645, 0.6566, 0.6751, 0.6748, 0.8375, 0.7696, 0.6753, 0.8391, 0.9836, 0.8851, 0.9120, 0.9113, 1.0483, 1.0773, 1.1733, 1.0596, 1.5733, 1.7083, 1.2570, 1.7263, 1.2766, 1.2985, 1.7630, 1.3211, 1.3503, 1.9316, 1.3551, 1.4595, 1.8808, 1.4880, 1.5728, 1.8881, 1.7460, 1.8275, 1.8375, 1.7746, 1.8503, 1.8878, 1.9558, 2.2100, 2.0408, 2.0903, 2.1093, 2.1330, 2.2460, 2.0048, 2.2878, 2.3203, 2.3513, 2.4951, 2.3470, 2.9911, 2.5260, 3.0256, 3.2678, 3.4045, 3.4846, 3.7433, 3.9143, 3.7455, 4.8073, 5.5295, 5.4005, 6.5541, 5.4435, 9.0960.

Table 2 displays certain descriptive statistics regarding set of observations under consideration which connotes that the data set is skewed and right tailed. In other words, since arithmetic mean is greater than 2^*th*^ quantile (median), which indicates that distribution is positive skewed. In addition to, the average life of fatigue fracture is 1.96 (± 1.57 Std.Dev) Kevlar 373/epoxy at constant pressure at the 90% stress level until all had failed while the maximum life of fatigue fracture is 2.29 Kevlar 373/epoxy.

**Table 2:** Descriptive statistics of life of fatigue fracture data.

Min.	Q1	Q2	Mean	Q3	Max.	Std.Dev	Skewness	Kurtosis
0.025	0.9048	1.7362	1.959	2.296	9.096	1.574	1.979	8.161

To demonstrate the efficacy of proposed model, we compare the HL-TEXPD with half-logistic Poisson (HLP) ([30]), type I half-logistic Bur-X (TIHL-BX) ([6]), type I half-logistic Frechet (TIHL-Fr) ([31]), Weibull-Truncated Exponential (W-TEXPD) ([32]), Truncated Exponential (TEXPD) and Exponential distributions.

It will be observed from the values of the parameter estimate and also the values of the criterion for comparison, the model that contains the minimum information loss which corresponds to minimum log-likelihood function ($\hat{l}$), AIC and BIC is considered to be the best model in the class of models considered. HL-TEXPD holds lowest numerical values of $\hat{l}$, AIC and BIC in Table (3) for certain data set than the competing models, which connotes that proposed model is better for describing the fatigue fracture data.

**Table 3:** Log-likelihood function ($\hat{l}$) evaluated at the MLEs of model parameters, the corresponding SEs (given in parentheses) and the statistics AIC and BIC.

Model	Estimates					Statistics	
	$\hat{l}$	$\hat{a}$	$\hat{\alpha }$	$\hat{\beta }$	$\hat{\theta }$	*AIC*	*BIC*
**HL-TEXPD**	**-122.67**	**0.025**	**—**	**—**	**0.735** (0.070)	**247.35**	**249.68**
HLP	-127.15		0.0359 (0.077)	—	28.4939 (2.325)	258.29	262.95
TIHL_*BX*_	-122.71		0.0135 (0.083)	0.556 (0.028)	76.239 (0.828)	251.42	258.41
TIHL_*Fr*_	-125.11		64.8083 (5.037)	0.3223 (0.281)	32.1303 (1.024)	256.23	263.22
W-TEXPD	-123.92	0.025	0.111 (0.341)	1.217 (0.108)	0.054 (0.166)	253.833	260.83
TEXPD	126.14	0.025	— (0.059)	—	0.517	254.270	256.601
Exponential	-127.11	—	0.510 (0.058)	—	—	256.23	258.56

**Fig 4:**
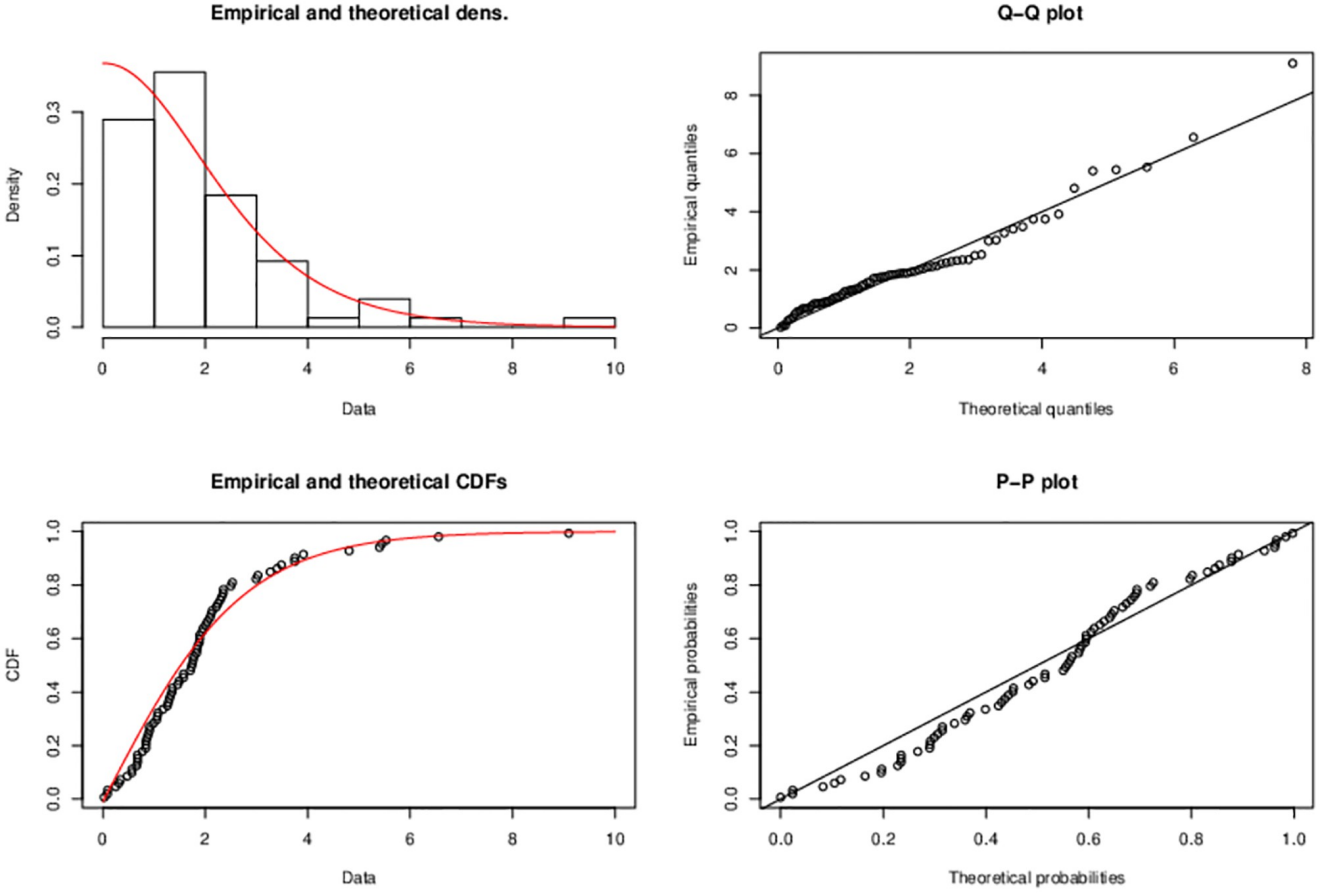
Plots of the (i) histogram and estimated PDF, (ii) quantile-quantile (Q-Q) (iii) empirical and estimated CDF, (iv) probability-probability (P-P) plot is a graph of the empirical CDF values plotted against the theoretical CDF values of Half Logistic-TEXPD model for first data.

The graphs in Fig 4 geometrically gives evidence that proposed model better fits the data.

### 4.2 Application 2: Actuarial science

The second data set is taken from [33] which represents survival time of seventy-two infected guinea pigs virulent with tubercle bacilli.

The data are as follows:0.1, 0.96, 0.56, 0.72, 0.44, 0.77, 0.93, 0.33, 0.59, 0.92, 1.00, 1.02, 1.00, 1.07, 0.70, 0.08, 1.08, 1.05, 1.08, 1.12, 1.09, 1.13, 0.74, 1.15, 1.16, 1.20, 1.21, 1.22, 1.22, 1.24, 1.3, 1.34, 1.36, 1.39, 1.44, 1.97, 1.46, 1.59, 1.72, 1.60, 1.68, 1.63, 1.71, 1.63, 1.76, 1.96, 1.83, 1.53, 1.95, 2.02, 2.15, 2.93, 2.16, 2.30, 2.53 2.31, 2.40, 2.51, 2.22, 2.54, 2.45, 2.54, 2.78, 2.13, 3.61, 3.27, 3.47, 3.42, 4.02, 4.58, 4.32, 5.55. The data is also analysed by [34].

Table 4 shows descriptive statistics regarding set of observations under consideration which connotes that the data set tends towards fat-tail. It can be defined as “*A fat-tailed distribution is a probability distribution that exhibits a large skewness or kurtosis, relative to that of either a normal distribution or an exponential distribution*”. It is worthy to mention that every fat-tailed distribution is heavy tailed but not vice versa, for instance, the Weibull distribution is heavy-tailed but not fat-tailed.

**Table 4:** Descriptive statistics of survival time of infected guinea pigs data.

Min.	Q1	Q2	Mean	Q3	Max.	Std.Dev	Skewness	Kurtosis
0.080	1.077	1.495	1.749	2.240	5.550	1.055	1.256	4.811

To demonstrate the efficacy of proposed model, we compare the HL-TEXPD with type-II Half-Logistic Weibull (TIIHLW) introduced by [35], Weibull-Truncated Exponential (W-TEXPD), Truncated Exponential (TEXPD) and Exponential distributions. Table (5) gives the numerical values of $\hat{l}$, AIC and BIC for model parameters. These statistics indicate good agreement between the HL-TEXPD and data set as compare to other fitted models.

**Table 5:** Log-likelihood function evaluated at the MLEs of model parameters, the corresponding SEs (given in parentheses) and the statistics AIC and BIC.

Model	Estimates					Statistics	
	$\hat{l}$	$\hat{a}$	$\hat{\alpha }$	$\hat{\beta }$	$\hat{\theta }$	*AIC*	*BIC*
**HL-TEXPD**	**-102.52**	**0.080**	**0.869** (0.083)	**—**	**—**	**207.05**	**205.33**
TIIHLW	-102.58	—	1.668 (0.671)	1.411 (0.051)	0.379 (0.009)	211.16	217.99
W-TEXPD	-101.19	0.080	0.012 (0.003)	1.496 (0.139)	0.006 (0.002)	208.38	215.21
TEXPD	-108.89	0.080	— (0.071)	—	0.599	219.78	222.05
Exponential	-112.26	—	0.572 (0.067)	—	—	226.52	228.79

**Fig 5:**
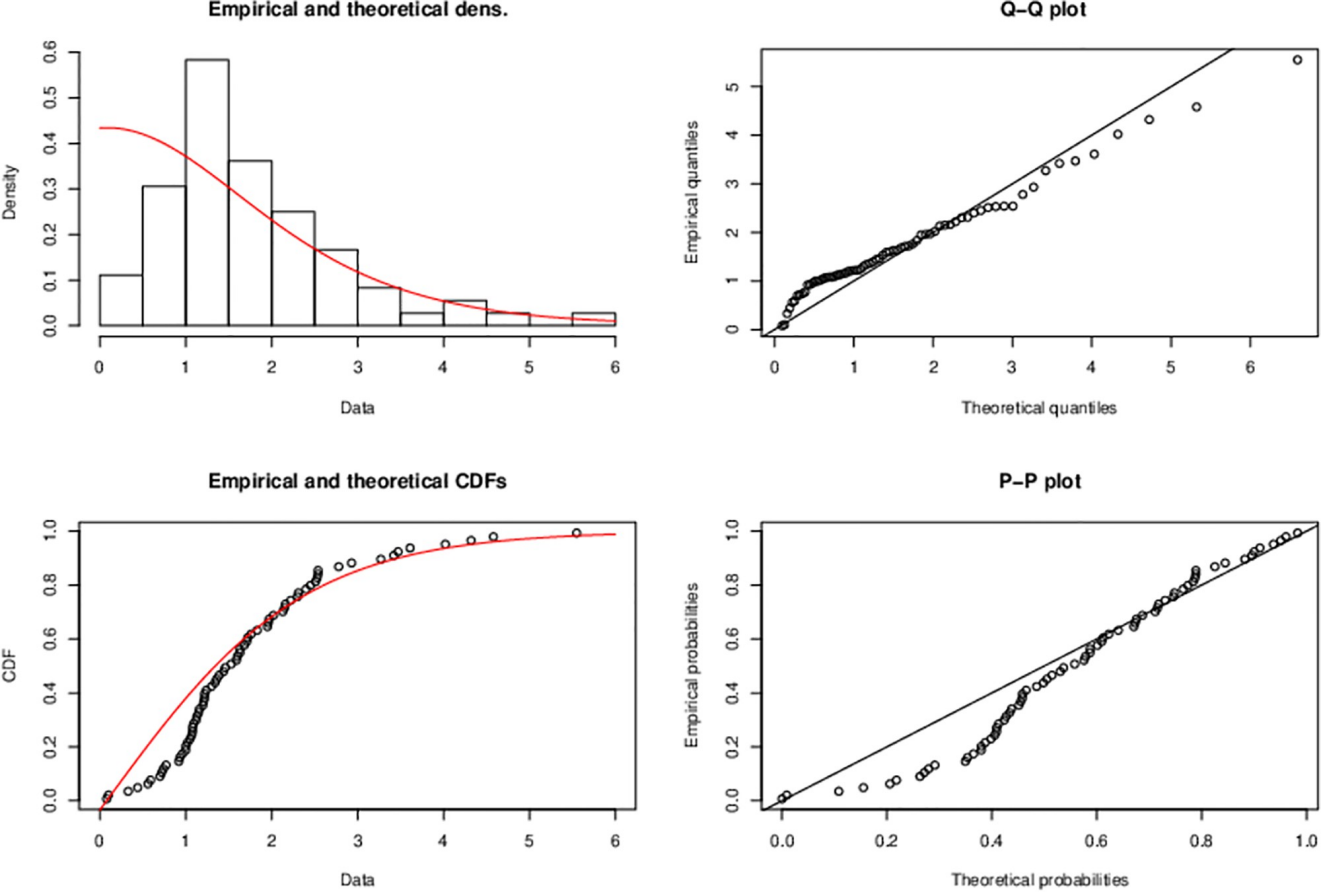
Plots of the (i) histogram and estimated PDF, (ii) quantile-quantile (Q-Q) (iii) empirical and estimated CDF, (iv) probability-probability (P-P) plot is a graph of the empirical CDF values plotted against the theoretical CDF values of Half Logistic-TEXPD model for second data.

The sketch in Fig 5 also supports the HL-TEXPD.

### 4.3 Application 3: Mechanical engineering

The third data set is published a manuscript [36]. The data are generated to test the performance of ball bearings and recorded the number of revolutions (in millions) before failure in life test. The readings are: 17.88, 41.52, 33.00, 42.12, 28.92, 48.80, 51.84, 45.60, 54.12, 67.80, 51.96, 68.64, 55.56, 68.88, 93.12, 68.64, 98.64, 84.12, 128.04 105.12, 127.92, 105.84, and 173.40.

Table 6 shows descriptive statistics regarding set of observations under consideration which connotes that data is positive skewed and Leptokurtic.

**Table 6:** Descriptive statistics for ball bearings data.

Min.	Q1	Q2	Mean	Q3	Max.	Std.Dev	Skewness	Kurtosis
17.88	47.20	67.80	72.24	95.88	173.40	37.479	0.941	3.488

To study the strength of proposed model, we compare the **HL-TEXPD** with Weibull-Truncated Exponential (W-TEXPD), Truncated Exponential (TEXPD) and Exponential distributions. The numerical values in Table 7 highlights that HL-TEXPD is best candidate for fitting the ball bearing data set as compare to rest of the three distributions.

**Table 7:** Log-likelihood function evaluated at the MLEs of model parameters, the corresponding SEs (given in parentheses) and the statistics AIC and BIC.

Model	Estimates					Statistics	
	$\hat{l}$	$\hat{a}$	$\hat{\alpha }$	$\hat{\beta }$	$\hat{\theta }$	*AIC*	*BIC*
**HL-TEXPD**	**-113.35**	**17.88**	**—**	**—**	**0.026** (0.004)	**228.70**	**229.83**
W-TEXPD	-114.17	17.88	-0.136 (0.037)	1.245 (0.215)	-0.002 (0.001)	234.34	237.74
TEXPD	-114.91	17.88	— (0.004)	—	0.018	231.81	232.95
Exponential	-121.43	—	0.014 (0.002)	—	—	244.88	246.01

**Fig 6:**
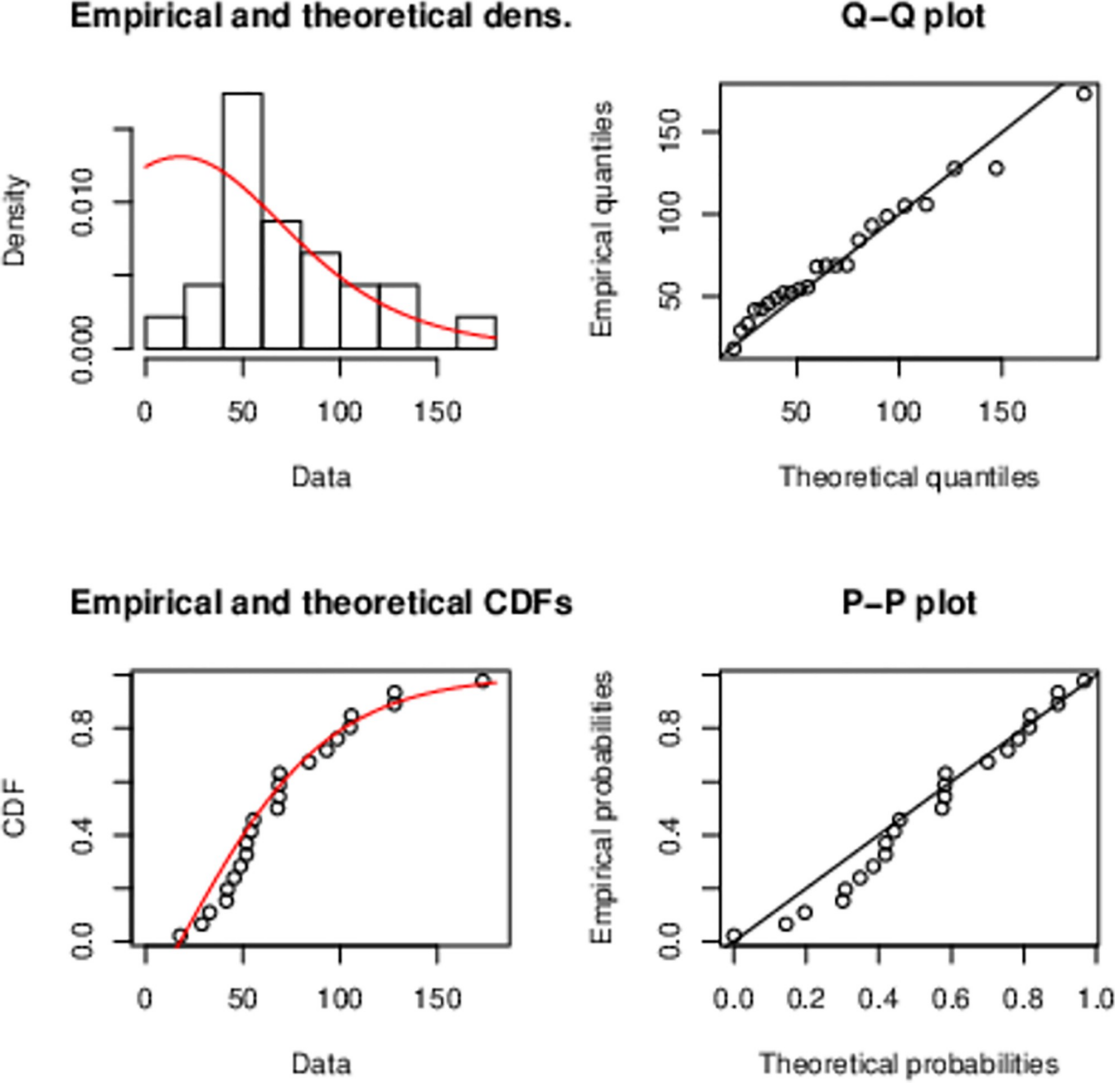
Plots of the (i) histogram and estimated PDF, (ii) quantile-quantile (Q-Q) (iii) empirical and estimated CDF, (iv) probability-probability (P-P) plot is a graph of the empirical CDF values plotted against the theoretical CDF values of Half Logistic-TEXPD model for third data.

Fig 6 suggests that HL-TEXPD better fits the data than other three classical models.

### 4.4 Exploratory data analysis

Exploratory data analysis written by [37] refers to the critical procedure of initial calculation of data to determine the pattern with help of summary statistics and graphical representation. It is an approach of statistical analysis that attempts to maximize insight into data. Exploratory data analysis uncovers underlying structure and extracts important variables of the data.

**Fig 7:**
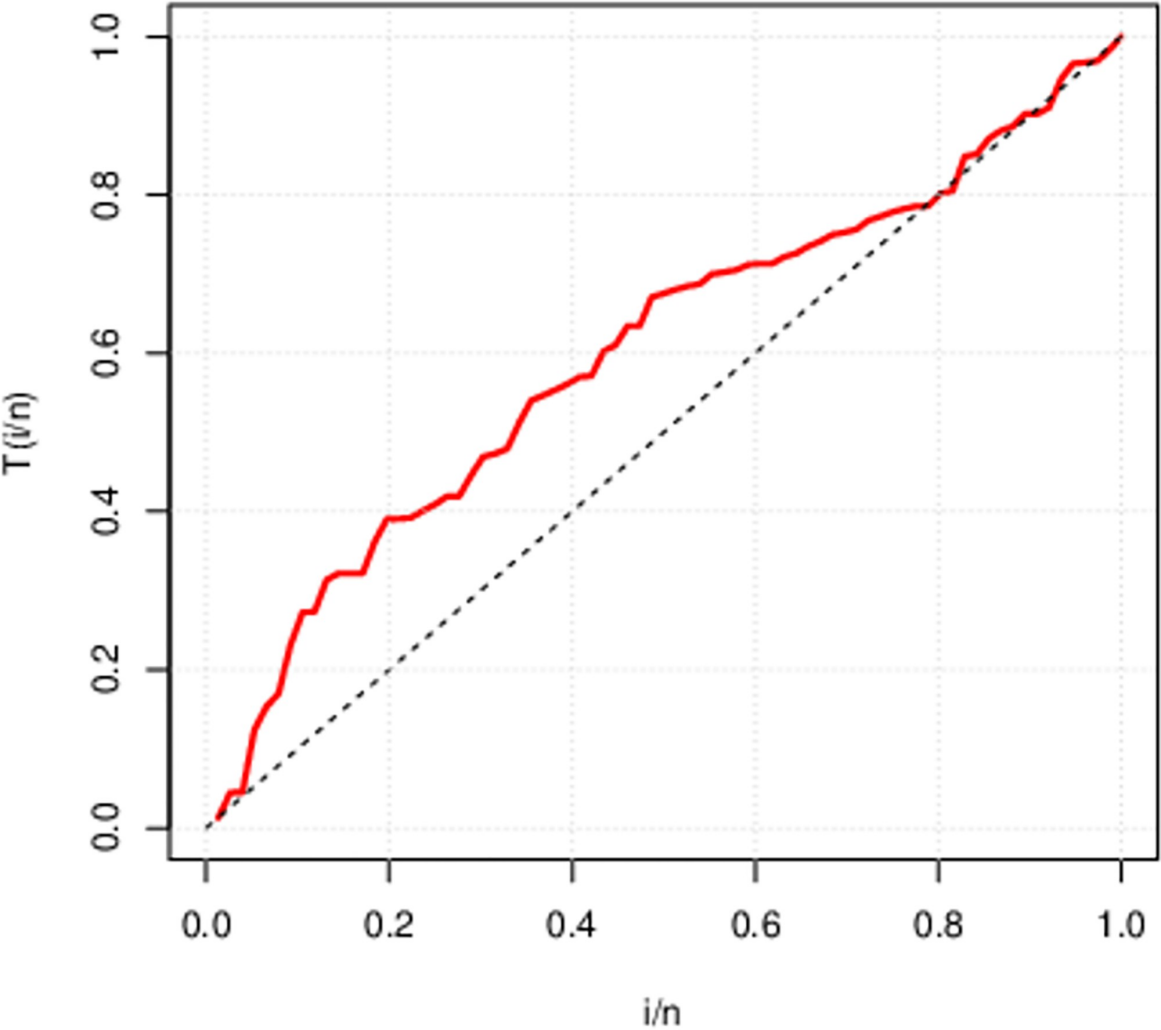
Total Time on Test (TTT) Plot for first data set (The life of fatigue fracture of Kevlar 373/epoxy at fixed pressure until all had failed).

**Fig 8:**
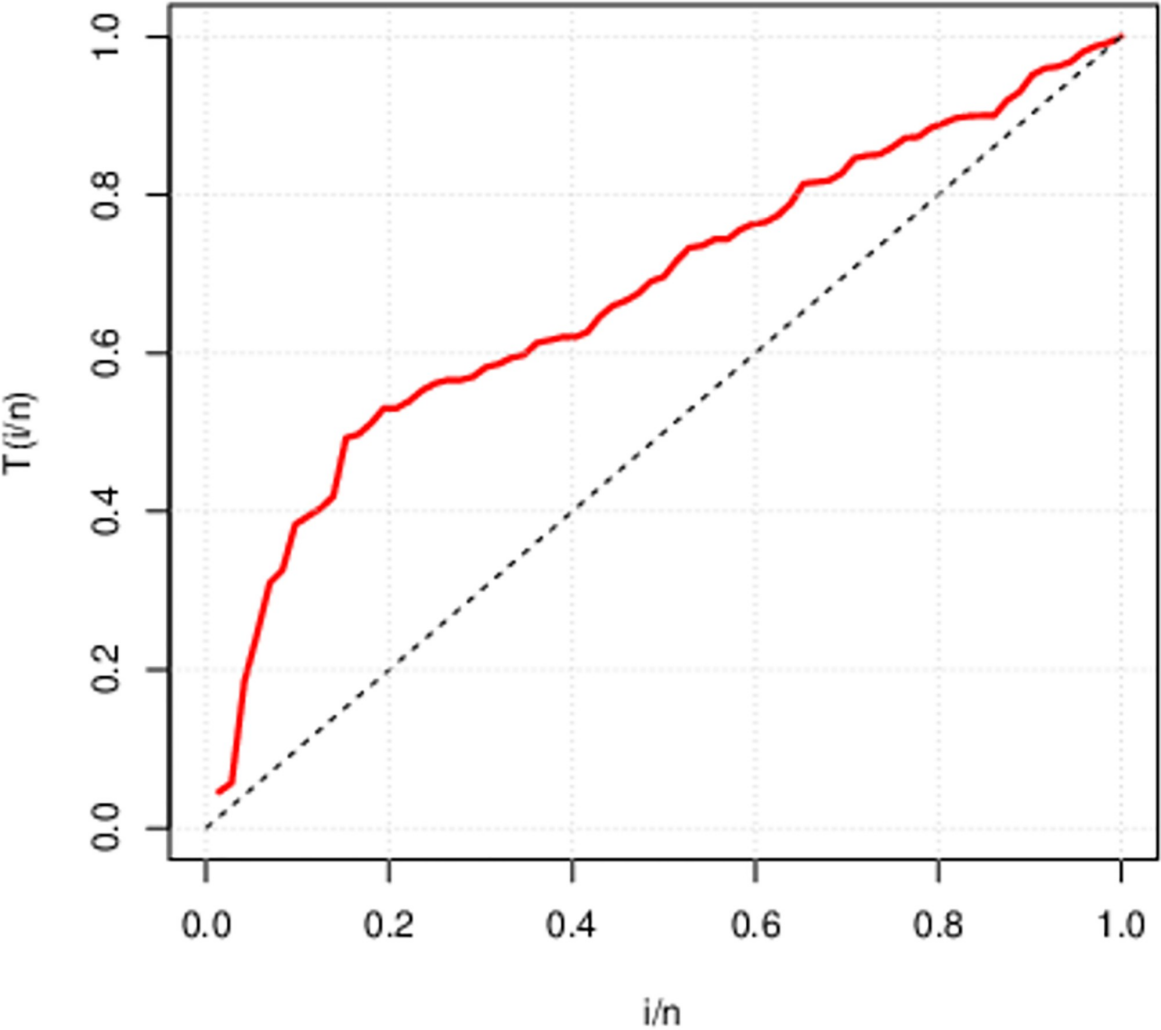
Total Time on Test (TTT) Plot for second data set (The survival time of seventy-two infected guinea pigs virulent with tubercle bacilli).

**Fig 9:**
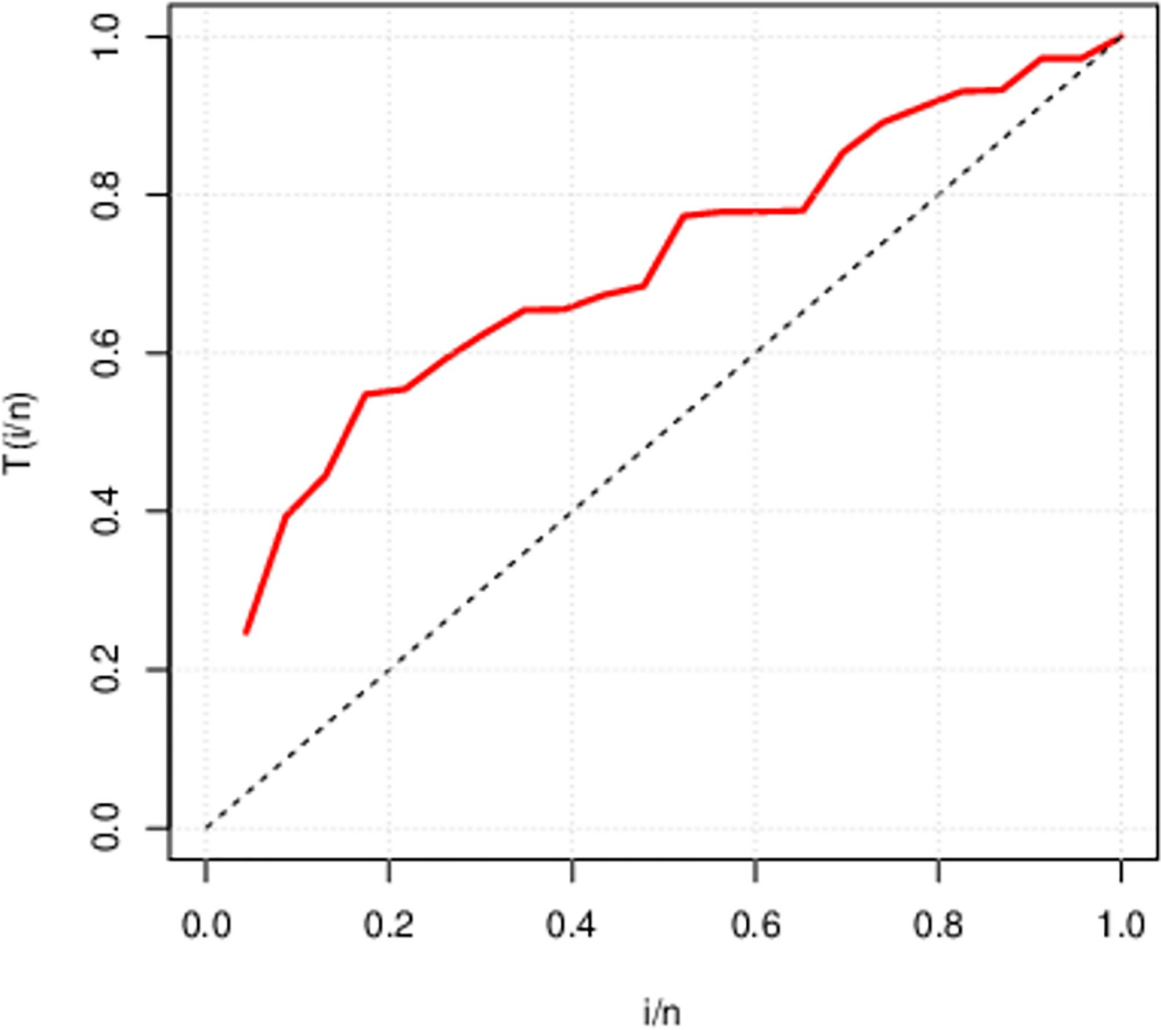
Total Time on Test (TTT) Plot for second data set (The performance of ball bearings and recorded the number of revolutions (in millions) before failure in life test).

The above Figs 7–9 are the TTT (total time on test) plot describing the failure rate of above three data sets. First data set portrays an upside down bath-tub failure rate. On the other side, the second and third data have concave shape indicate that both data sets possess an increasing failure rate. We can figure out that HL-TEXPD can be efficiently used to accommodate failure rates of the data having concave and bath-tub shapes.

## 5 Conclusion

[24] introduced a new modified form of renowned “Half logistic” distribution introduced by [1] so-called Half-logistic Truncated Exponential distribution (HL-TEXPD). Some mathematical characteristics of HL-TEXPD are studied. HL-TEXPD is fitted on three real data sets. The results verdict that HL-TEXPD better fit the data sets under study as contrary to rest of the classical and baseline models. The TTT plot is fitted to examine failure rate trend of the afore mentioned data sets. It is concluded upon the evidence of TTP plot that HL-TEXPD is useful to model the data having upside down bath-tub, concave shape or increasing failure rate function, thus these characteristics would encourage the researchers to analyze the engineering and lifetime data by using the proposed model. The Monte Carlo simulation of HL-TEXPD is conducted for different combinations values of the parameter for different sample sizes to study the average ML estimates, standard errors, biases, mean square errors, mean relative error and corresponding coverage probabilities at 99% confidence intervals. The simulation results connote that 99% coverage probability for approximate confidence intervals of true parameter based on MLEs give satisfactory results. A real life data sets from mechanical engineering and actuarial sciences are presented to compare the performance of our model with the truncated and un-truncated contemporary models. Several statistical criteria i.e. negative log-likelihood, AIC, BIC, K-Smirnov, C-Von and A-Darling are used to collect enough evidence for the better performance of HL-TEXPD. Since the proposed distribution having two parameters i.e. location and scale, thus in future, an additional shape parameter can be added to study the direction and control of outliers in the data sets. Furthermore, bivariate or multivariate version of HL-TEXPD can be developed to study the engineering and medical problems in different dimensions.
